# Microbiome determinants of productivity in aquaculture of whiteleg shrimp

**DOI:** 10.1128/aem.02420-24

**Published:** 2025-04-15

**Authors:** Xiaoyu Shan, Kunying Li, Patrizia Stadler, Martha Borbor, Guillermo Reyes, Ramiro Solórzano, Esmeralda Chamorro, Bonny Bayot, Otto X. Cordero

**Affiliations:** 1 Department of Civil and Environmental Engineering, MIT 122707 , Cambridge, Massachusetts, USA; 2 Centro Nacional de Acuicultura e Investigaciones Marinas, CENAIM, ESPOL, Escuela Superior Politécnica del Litoral 27883, Guayaquil, Ecuador; 3 Universidade Federal de Santa Catarina 28117, Florianópolis, Brazil; 4 Facultad de Ingeniería Marítima y Ciencias del Mar, FIMCM, ESPOL, Escuela Superior Politécnica del Litoral 27883, Guayaquil, Ecuador; INRS Armand-Frappier Sante Biotechnologie Research Centre, Laval, Quebec, Canada

**Keywords:** aquaculture, microbiomes, food

## Abstract

Aquaculture holds immense promise for addressing the food needs of our growing global population. Yet, a quantitative understanding of the factors that control its efficiency and productivity has remained elusive. In this study, we address this knowledge gap by focusing on the microbiome determinants of productivity, more specifically animal survival and growth, for one of the most predominant animal species in global aquaculture, whiteleg shrimp (*Penaeus vannamei*). Through analysis of the shrimp-associated microbiome from previous studies across Asia and Latin America, we established the presence of core phylogenetic groups, widely prevalent across aquaculture conditions in disparate geographic locations and including both pathogenic and beneficial microbes. Focusing on the early stages of growth (larval hatcheries), we showed that the composition of the microbiome alone can predict a remarkable fraction of the variation in shrimp larvae survival rates (ca. 50%). Taxa associated with high survival rates share recently acquired genes that appear to be specific to aquaculture conditions. These genes are involved in the biosynthesis of growth factors and protein degradation, underscoring the potential role of beneficial microorganisms in nutrient assimilation. By contrast, the predictability of the microbiome on the adult shrimp weight in grow-out farms is weaker (10%–20%), akin to observations in the context of livestock. In conclusion, our study unveils a novel avenue for predicting productivity in shrimp aquaculture based on microbiome analysis. This paves the way for targeted manipulation of the microbiome as a strategic approach to enhance aquaculture efficiency from the earliest developmental stages.

**IMPORTANCE:**

Aquaculture is a rapidly growing industry essential for global food security, yet its productivity is often constrained by high mortality rates and inefficient growth. While the microbiome is known to influence host health and nutrient assimilation, its broader role in animal production remains poorly understood. Here, we take a data-driven approach to address this gap by systematically analyzing shrimp-associated microbiomes across hatcheries and farms. By integrating microbiome data with machine learning, we demonstrate that microbial communities are powerful predictors of key production outcomes, shaping shrimp survival and growth. Our findings suggest that the microbiome could serve as a diagnostic tool for assessing production conditions and optimizing management strategies. In addition, machine learning techniques offer a promising avenue for identifying beneficial microbes and developing targeted microbiome therapies to enhance aquaculture sustainability and efficiency.

## INTRODUCTION

In aquaculture and animal farming, in general, animal survival and individual growth yield are key factors that affect global measures of performance (1–3). Improving resilience against disease (i.e., survival) and the efficiency with which animals assimilate the feed (individual growth yield) lies at the heart of addressing the twin challenges faced by the future food systems: elevating production to satisfy the escalating global food demand while reducing environmental impacts to align with sustainable development goals. Within this framework, the host microbiome is widely acknowledged to play an important role (4–9). Beneficial microbes have the potential to improve survival and growth by facilitating the digestion and assimilation of nutrients (10) or safeguarding against diseases (11). Conversely, pathogenic microbes may compromise production by hindering growth or increasing mortality (12), thus reducing yield in relation to resource consumption.

Since the late 1980s, aquaculture has witnessed a rapid increase, in contrast to the stable production of marine capture fisheries, providing high-quality edible proteins and setting a new record with earnings of USD 265 billion (only for aquatic animals) and employing 20.7 million people as of 2020 (13). Whiteleg shrimp (*Penaeus vannamei*) farming, in particular, leads in aquaculture animal production with an annual production of 5.8 million tons in 2020 (13). Despite its rapid expansion, the productivity of shrimp aquaculture frequently faces setbacks due to feed wastage (14) and pathogen infections (15), underscoring the need for technological advancements to improve production through microbiome engineering. However, the variable outcomes associated with probiotic use in shrimp farming have generated both enthusiasm and skepticism (16, 17). This highlights a significant gap in our understanding caused by a lack of comprehensive, systematic studies on how the microbiome composition affects productivity in shrimp aquaculture. Addressing this gap is crucial for leveraging microbiome-based strategies to their fullest potential.

In this study, we investigate the linkage between the microbiome and production variables in the aquaculture of *P. vannamei*. We start by characterizing a shrimp-associated microbiome across hatcheries and farms distributed across the globe. Subsequently, we focus separately on the two major phases of shrimp production: the hatchery phase and the grow-out phase. In the hatchery phase where shrimp transition from nauplii to postlarvae, survival rates are critical for productivity as early-stage shrimp are particularly vulnerable to environmental stressors and pathogenic threats. We investigate how the microbiome influences these survival rates, taking advantage of the tightly controlled abiotic conditions in hatcheries. In the grow-out phase, where the major goal is achieving optimal growth as shrimp advance to marketable sizes, we focus on the body weight of shrimp as a direct indicator of growth efficiency and productivity under varying conditions of water quality and nutrient availability. By identifying prevalent taxa and key functional genes across global farms and evaluating the predicting power of microbiomes at different phases of shrimp production, we aim to shed light on the potential of microbiome interventions in enhancing the sustainability and productivity of shrimp aquaculture.

## RESULTS

### Characterizing the global shrimp-associated microbiome

**Fig 1:**
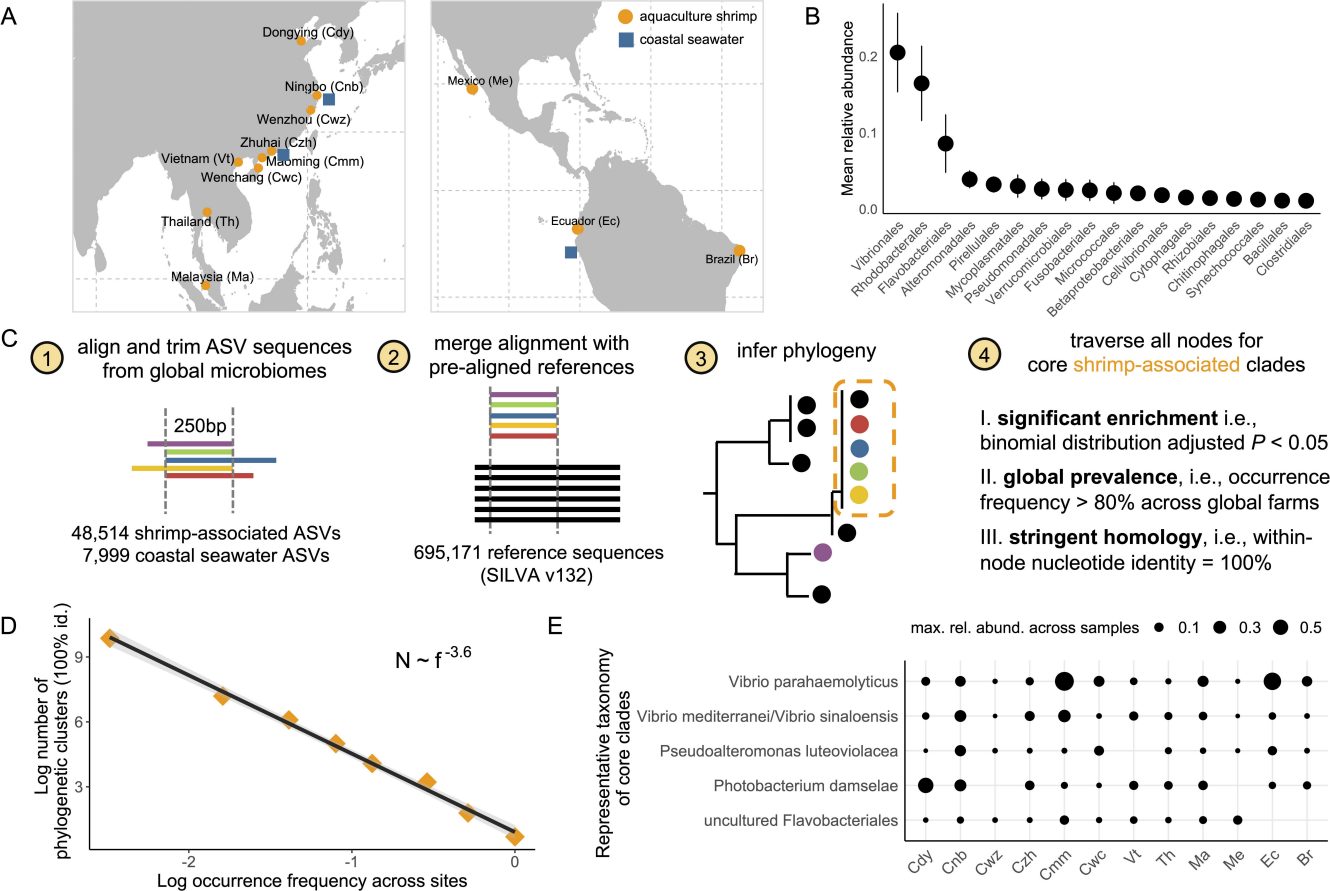
Identifying core clades of shrimp-associated microbiome from global aquaculture. (**A**) Geographic distribution of microbiome data sets compiled in this study. The orange circle indicates a shrimp-associated microbiome. Blue square indicates coastal seawater microbiome. (**B**) Ranked mean relative abundance of bacterial taxa across global shrimp-associated microbiomes. (**C**) A bioinformatic pipeline is developed to identify core clades of shrimp-associated microbiome. See Materials and Methods for full descriptions. (**D**) A power law relationship between the number of phylogenetic clusters N and the occurrence frequency of phylogenetic clusters f. (**E**) Representative taxonomy of the identified core clades of global shrimp-associated microbiome. The size of the dots indicates the maximal relative abundance of a clade in a microbiome. Acronyms of locations are provided in panel **A**. Cdy, China Dongying; Cnb, China Ningbo; Cwz, China Wenzhou; Czh, China Zhuhai; Cmm, China Maoming; Cwc, China Wenchang; Vt, Vietnam; Th, Thailand; Ma, Malaysia; Me, Mexico; Ec, Ecuador; Br, Brazil.

To better understand the role of the microbiome on shrimp production, we first compiled a global data set of aquaculture shrimp-associated microbiome (Method). Briefly, we re-analyzed microbiome sequencing data of 579 samples (18–27) from 12 geographically distant shrimp cultures in Asia and Latin America, including 231 samples of shrimp larvae microbiome and 348 samples of intestine (gut) microbiome from juvenile or adult shrimp (Fig. 1A; Table S1). Recognizing the coastal ocean as the natural source of microbes in marine aquaculture, we also incorporated 154 samples of coastal seawater microbiome (28–30). This extensive effort yielded a database of 56,513 ASVs (Amplicon Sequence Variants), including 48,514 shrimp-associated ASVs and 7,999 coastal seawater ASVs, now publicly available for further aquaculture research.

With this data set of global shrimp-associated microbiomes, we begin by asking whether the aquaculture environment selects for specific taxonomic clades or simply reflects a random assortment from the surrounding seawater. We find that the mean pairwise phylogenetic distances (MPD) between shrimp-associated sequences and coastal seawater sequences are significantly higher than that expected by a null model (Nearest Relatedness Index = 9.7, *P* < 0.001, see Materials and Methods), suggesting a selective adaptation of specific microbes to a farm-associated environment. Indeed, we identified taxonomic groups that are significantly enriched or diluted in shrimp-associated microbiomes. Oligotrophic taxa such as SAR11 and SAR86 are significantly less prevalent (Fig. S1, adjusted *P* < 0.01), while copiotrophic taxa such as Vibrionales, Rhodobacterales, and Flavobacteriales are among the most abundant ones across global aquaculture shrimp microbiome (Fig. 1B). Interestingly, these taxa were also among the most abundant taxa in microbial communities assembled on marine particles of polysaccharides such as chitin (31, 32). Vibrionales are known for their fast growth rate, especially in saline and estuarine environments (0.5–3% NaCl) and high temperature (25–30°C) featured by aquaculture conditions (33). Flavobacteriales are noted for their capabilities in degrading high-molecular-weight polysaccharides and proteins (34–36), whereas Rhodobacterales are specialized in scavenging metabolic byproducts such as organic acids and amino acids (34, 35).

Having elucidated the overall taxonomic composition of shrimp-associated microbiome, we further ask if there are core clades at finer phylogenetic scales that are conserved and significantly enriched across global shrimp cultures (Fig. 1C, see Materials and Methods). By grouping ASV ribotypes into phylogenetic clusters, we identify a power law relationship between the number of phylogenetic clusters (*N*) and their occurrence frequency (*f*): $N\sim f^{-a}$, with a=-3.6 (Fig. 1D). This power law exponent is larger in magnitude than those observed in other systems (37), including the global ocean microbiome (where a = −1.7) (38). This indicates a more rapid decline in the occurrence frequency as we move from the most to the least ubiquitous cluster, suggesting that the microbiome of different shrimp farms is dominated by a few nearly ubiquitous clusters (Fig. 1E). These clusters include three clades related to Vibrionales, represented by *Vibrio parahaemolyticus*, *Vibrio mediterranei*/*sinaloensis,* and *Photobacterium damselae*, all of which are commonly known as aquaculture pathogens of fish or shrimp (39–41). In particular, *V. parahaemolyticus* (Vp), the causative agent of acute hepatopancreatic necrosis disease (AHPND), is notorious for causing enormous economic losses to global shrimp farms every year (12). In addition to these pathogens, a clade in the order of Alteromonadales, represented by *Pseudoalteromonas luteoviolaceae*, is also part of the core shrimp farm microbiome. Members of this clade have been shown to inhibit shrimp pathogens by producing antimicrobial compounds (42), suggesting that some of the core microbiome members may confer beneficial effects to the host.

### Microbiome composition predicts survival in shrimp larvae hatcheries

**Fig 2:**
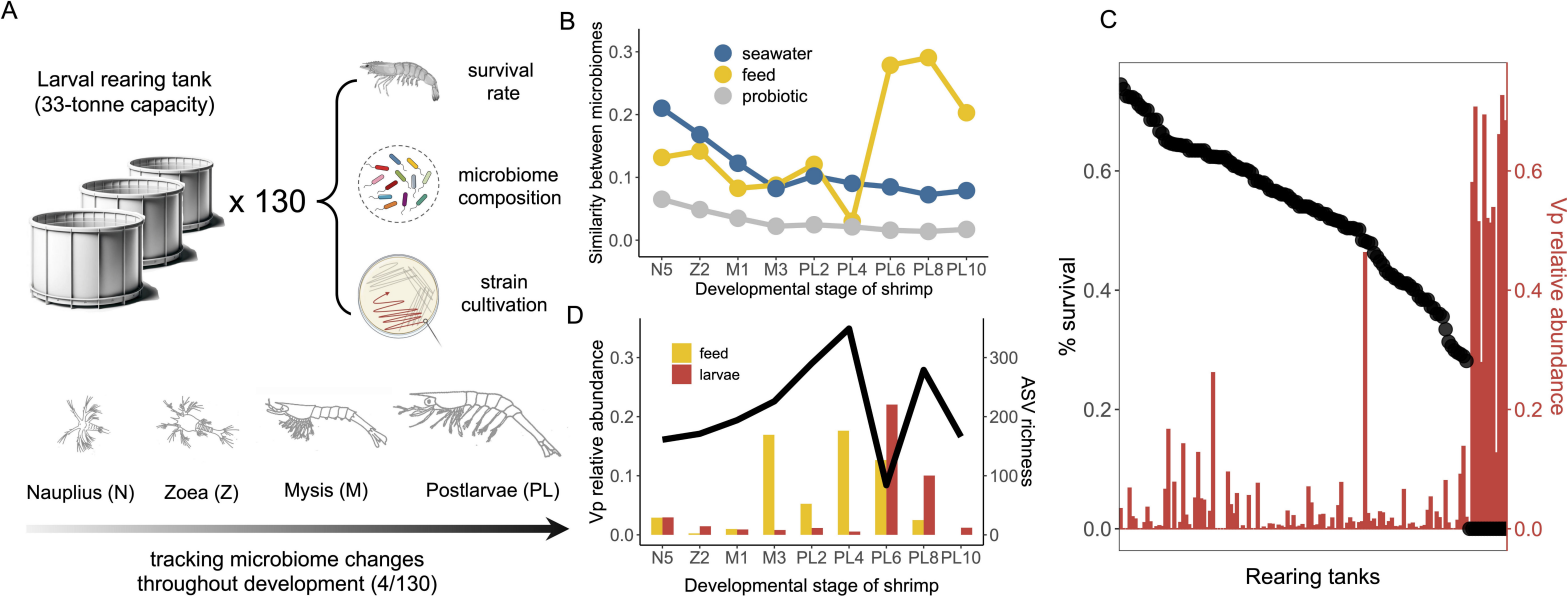
Microbiome characterization in a shrimp hatchery. (**A**) Shrimp larvae were sampled from 130 33-ton capacity tanks where the survival rate of shrimp larvae was counted and recorded. The sampled shrimp larvae were further used for microbiome sequencing and bacterial strain isolation. For four selected tanks, we sampled throughout the developmental stages from nauplius, zoea, and mysis to postlarvae. The potential sources of microbiome such as seawater, feed (microalgae at the nauplius and zoea stage and *Artemia* at the mysis and postlarvae stage) as well as probiotics were also sampled and sequenced. (**B**) The similarity between shrimp larvae-associated microbiome composition and microbiome composition of potential sources. Similarity is calculated as 1- Bray-Curtis dissimilarity based on relative abundance. (**C**) Black dots show the ranked survival rate of shrimp larvae across 130 tanks. The red bar shows a relative abundance of *V. parahaemolyticus* in the corresponding shrimp larvae-associated microbiome. (**D**) The black line indicates the richness of shrimp larvae-associated microbiome across developmental stages. The red bar and yellow bar indicate relative abundances of *V. parahaemolyticus* across developmental stages in shrimp larvae microbiome and microbiome in the feed. Icons of shrimp and petri dish were created by BioRender.

We first probe into the impacts of microbiome composition on productivity at the hatchery stage. Here, shrimp larvae are nurtured from their earliest nauplii (N) stage to the postlarvae (PL) phase, preparing them for transfer to large-scale farms. We tracked the microbiome succession through developmental stages (nauplii, zoea, mysis, and post-larvae) in a shrimp hatchery over a period of 18 days (Fig. 2A, see Materials and Methods). Initially, the microbiome composition of the larvae was most similar to the surrounding seawater, especially during the early nauplii and zoea stages (Fig. 2B). This resemblance gradually diminishes as the larvae mature toward the PL stage (from ~30% shared ASVs to~8% shared ASVs, ~20% compositional similarity to ~10% compositional similarity). At this stage, the larvae-associated microbiome became most similar to that of the larval feed, which was based on the aquatic crustacean *Artemia franciscana* (~40% shared ASVs, ~30% compositional similarity; Fig. 2B; Fig. S2). Such opposite trends of microbiome similarities with seawater and feed indicate a transition of microbial influences from environmental to dietary sources. Interestingly, the bacteria administered through probiotics (>80% composed of *Lactobacillaceae*) appear to be unable to colonize the host. The relative abundance of *Lactobacillaceae* never reached above 1% throughout all larval developmental stages. This result highlights the need to develop new microbiome therapies based on microorganisms native to the conditions of seawater aquaculture.

To map microbiome composition to productivity, we sampled extensively across 130 tanks of 33-ton capacity from this hatchery (Fig. 2A, see Materials and Methods). These tanks represent well-controlled replicate environments that undergo identical feeding regimes; therefore, the differences in animal survival represent a major cause of variability in productivity and feed efficiency. Consistent with observations of Vp causing large-scale losses in production, we found in this hatchery that 10% (13 out of 130) of tanks showed signs of collapse (no harvest) and that the microbiome of the larvae in these tanks was made of over 50% Vp (Fig. 2C). Our microbiome development data showed that the rise of Vp in the larval microbiome was a sudden event, with an increase from nearly zero to ~20% relative abundance from day 4 to day 6 at the PL stage (Fig. 2D). The observed surge in Vp abundance first appeared in the feed (i.e., Artemia) before being mirrored in the larvae (Fig. 2D; Fig. S3), aligning with the developmental phase when the larvae microbiome is primarily shaped by their feed rather than the ambient seawater. This pattern indicates that *Artemia* can be a primary vector for Vp transmission. Interestingly, excluding these collapsed tanks, the remaining 90% of tanks had variable larvae survival rates, from ~30% to ~80% (Fig. 2C). These rates were only poorly correlated to Vp’s abundance (Pearson’s r = 0.007, *P* = 0.98), indicating that other microbiome components may instead determine productivity. Variation in these rates could not be attributed to differences in the alpha diversity of the microbiome (Fig. S4).

**Fig 3:**
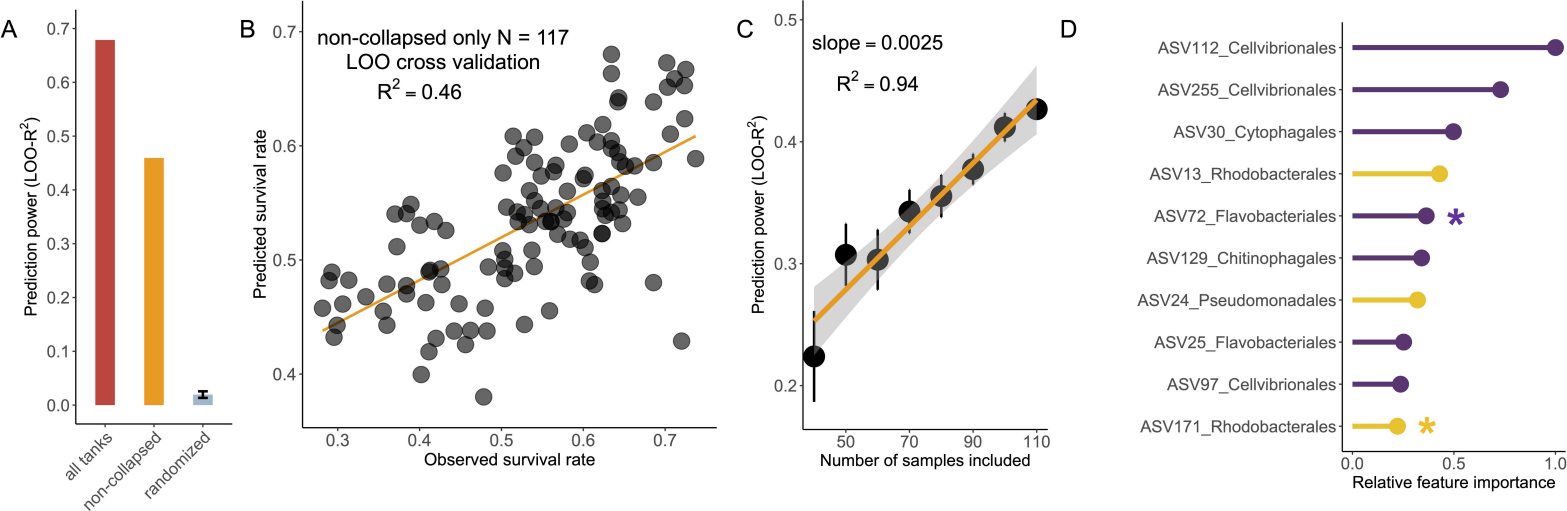
Microbiome composition as a strong predictor of shrimp larvae survival. (**A**) Statistical learning of microbiome composition to predict survival rate of shrimp larvae. Prediction power is indicated by leave-one-out cross-validation R^2^. (**B**) The observed survival rate of shrimp larvae and that predicted by microbiome composition through leave-one-out cross-validation. The 13 tanks where a bloom of *V. parahaemolyticus* led to the collapse of all shrimp larvae were excluded. (**C**) The prediction power of microbiome composition linearly increases as the number of samples increases. On average, increasing every 10 samples leads to a 2.5% increase in prediction power. (**D**) Taxa show the highest feature importance in statistical learning to predict the survival rate of shrimp larvae. Purple lines indicate potential primary degraders. Yellow lines indicate potential scavengers.

Given this large variation in larvae survival across tanks, we asked to what extent it can be attributed to differences in microbiome composition. To address this question, we employed a Random Forest model to predict the survival rate of shrimp larvae based on their microbiome composition. We utilized a leave-one-out (LOO) cross-validation approach, allowing us to maximize the use of all available samples while ensuring an independent test set to prevent overfitting. With all samples, we find that microbiome composition can predict nearly 70% of the observed variation in larvae survival (LOO-R^2^ = 0.68, Fig. 3A), which is as expected since a single ASV of Vp is known to be a strong indicator. We then ask how predictable the survival rate is if we exclude all the collapsed tanks. Remarkably, we found that microbiome composition alone still predicts nearly 50% of the variation in survival rate (LOO-R^2^ = 0.46, Fig. 3B). To test the robustness of this prediction, we conducted a series of random tests by reshuffling the survival rates across tanks 100 times and reassessing predictability of the model. These randomization tests consistently resulted in a significantly reduced predictive power (average LOO-R^2^ <0.05, Fig. 3A), demonstrating that specific microbiome compositions are indeed closely linked to survival outcomes in shrimp larvae, instead of due to chance. We further show that the prediction power of the model linearly increases with the number of samples (2.5% net increase in LOO-R^2^ per 10 samples added, linear regression R^2^ = 0.94, *P* < 0.001, Fig. 3C), suggesting that the predictability of larvae survival by microbiome composition can be further elevated by expanding the sample space (i.e., sampling more tanks).

**Fig 4:**
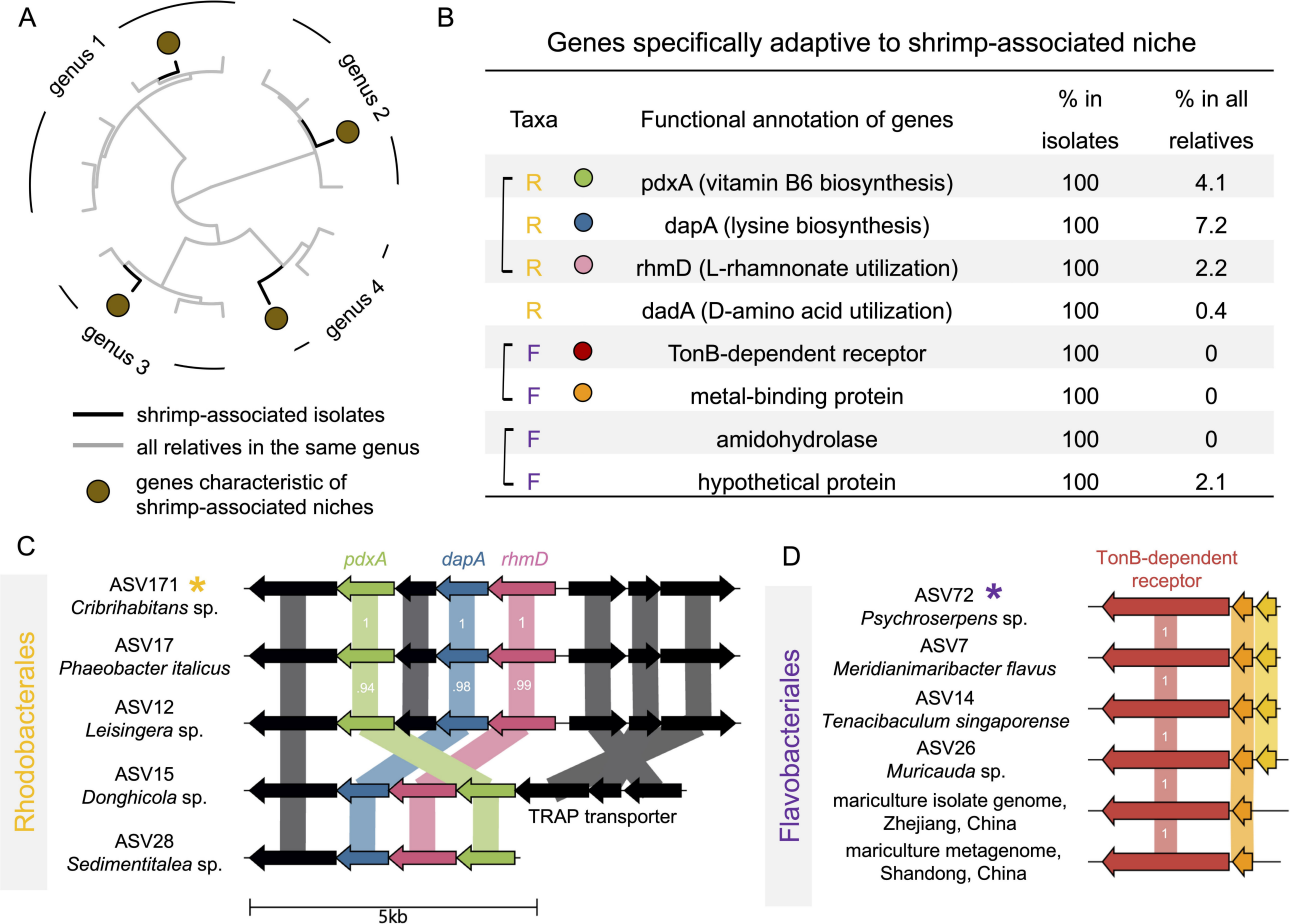
Horizontal transfer of genes that are specifically adaptive to a shrimp-associated niche. (**A**) Schematic illustration of comparative genomic analysis for identifying genes that are specifically adaptive to a shrimp-associated niche. The selected genes are shared by isolates that are abundant in the shrimp larvae microbiome but are present in none or very few (<10%) of all the other publicly available genomes in the same taxonomic clades. Tips colored in black indicate strains isolated from the shrimp larvae samples. Tips colored in gray indicate publicly available genomes in the same genus as the shrimp-associated isolates. The brown circle indicates a gene that is only present in the genome of shrimp-associated isolates but not in any other genomes from the same genus. (**B**) A total of 8 genes are identified from the shrimp larvae-associated microbiome. Genes marked with a yellow “R” are shared by all five abundant isolates in the Rhodobacterales clade. Genes marked with a purple “F” are shared by all four abundant isolates in the Flavobacteriales clade. All these genes are rarely present in other genomes of the same genera from public databases (<10%). The 16S rRNA sequences of all nine isolates are 100% identical to ASVs in the sequenced shrimp larvae microbiome whose maximal relative abundances across tanks are at least 1%. Square brackets indicate that genes are next to each other in the genome. (**C**) Gene map of adjacent genes (*pdxA*, *dapA,* and *rhmD*) experiencing horizontal transfer across five isolates from distinct genera in the order of Rhodobacterales. Numbers in the gene map indicate the level of amino acid sequence identity between homologs. (**D**) Gene map of two adjacent genes (TBDR and a metal-binding protein gene) experiencing recent horizontal transfer among four isolates from distinct genera in the order of Flavobacteriales isolated from shrimp larval hatchery in Ecuador, as well as their homologs in publicly available genome and metagenome derived from aquaculture in different regions of China. All the six TBDR genes have 100% identical sequences with each other.

We aimed to gain a deeper understanding of the taxa that play a key role in predicting larvae survival, especially their ecological roles and genomic features. We calculated the relative feature importance of each ASV, finding that a majority of the top important ones (7 out of top 10) are from taxonomic groups well known as polysaccharide degraders (35) (e.g., Cellvibrionales, Cytophagales, and Flavobacteriales, Fig. 3D). These microorganisms often encode various carbohydrate-active enzymes (CAzymes), potentially contributing to polysaccharide degradation utilization in the aquaculture system (36). Moreover, among the top important taxa are two ASVs from the Rhodobacterales clade, whose members have been reported to produce metabolites antagonistic to Vibrio pathogens (43, 44). To better understand the genomic features of these important taxa, we cultivated a total of 401 strains from the larvae samples, out of which 2 strains had 100% 16S rRNA gene sequence identity with the top 10 important ASVs from our Random Forest model (Fig. 3D; Fig. S6), one in the Rhodobacterales clade (ASV171, *Cribrihabitans* sp.) and the other in the Flavobacteriales clade (ASV72, *Psychroserpens* sp.). In addition to these two isolates, we isolated seven other strains within these two clades that are abundant (at least 1% relative abundances) in the shrimp larvae microbiome (Table S2; Fig. S6), suggesting that they may have fitness advantages in the shrimp larvae-associated environment. We therefore leverage the genome of these isolates (we refer to them as “abundant isolates” thereafter) to identify genes that are specifically adaptive to a shrimp-associated niche. Briefly, we filter for genes that are shared by *all* our shrimp-derived abundant isolates but are rarely present (<10%) across non-shrimp-derived close relatives (i.e., in the same genus with the shrimp-derived abundance isolates) (Fig. 4A, see Materials and Methods for full details). This led to the discovery of recent horizontal gene transfer events involving these abundant shrimp larvae-derived strains in the Rhodobacterales or Flavobacteriales clades (Fig. 4B and C, see Files S1 and S2 for sequences). Among the Rhodobacterales strains, we observed recent horizontal transfer of genes involved in the biosynthesis of growth factors, such as vitamin B6 (45) and lysine (46) (Fig. 4B and C, 100% nucleotide identity between homologs in *Cribrihabitans* sp. ASV171 and *Phaeobacter italicus* ASV17), suggesting a potentially beneficial role of nutritional supplementation for the host. Among the Flavobacteriales strains, we identified a recent horizontal transfer of amidohydrolase genes, crucial for protein degradation, as well as a gene encoding a TonB-dependent receptor (TBDR) (Fig. 4B and D, 100% nucleotide identity between homologs in *Psychroserpens* sp. ASV72 and *Meridianimaribacter flavus* ASV7). An exhaustive search of the public database further revealed homologs of this TBDR, such as an isolate genome in Zhejiang Province of China (*Maribacter aurantiacus* KCTC 52409, GCF_005780245.1), as well as environmental metagenomes in Shandong Province of China (ERR2094176), both showing 100% amino acid identity with the TBDR swept across our strains isolated from the shrimp hatchery in Ecuador (Fig. 4D). Both the Chinese genome and metagenome are also derived from seawater aquaculture, underscoring global ecological relevance of this gene that is specifically adaptive to the aquaculture environment. Despite the functional versatility of TBDR, previous work on marine microbiology has highlighted the important role of TBDR in polysaccharide transport for Flavobacteriales (34). Previous studies have also shown a TBDR with exactly the same domain organization (i.e., a carboxypeptidase regulatory-like domain along with an outer membrane channel) functions in collagen degradation in coral-reef-associated ecosystems (47), which could inspire further biochemical studies confirming the function of this TBDR.

### Water quality is a stronger predictor of adult shrimp growth than the microbiome

Having established the significant predictive power of microbiomes for animal survival in the hatchery phase, our investigation extends to the impact of microbiomes on the growth of adult shrimp in shrimp grow-out ponds. In contrast to the well-controlled conditions of hatchery tanks, adult shrimp grow in dugout ponds that extend over several hectares of water—at least 100 times larger than a hatchery tank. These are complex ecosystems that farmers manage on an individual basis to achieve maximal productivity. Despite the expected heterogeneity of these systems, we asked to what extent the microbiome of individual animals could predict the individual phenotypes, in particular, body weight. To this end, we sampled 226 adult shrimps across 6 grow-out ponds, recording the body weight of each individual. We sequenced the microbiome of the hepatopancreas for all sampled shrimps, considering that it is a primary focus of Vp infections, as well as the intestine microbiome for a subset of 76 shrimps.

**Fig 5:**
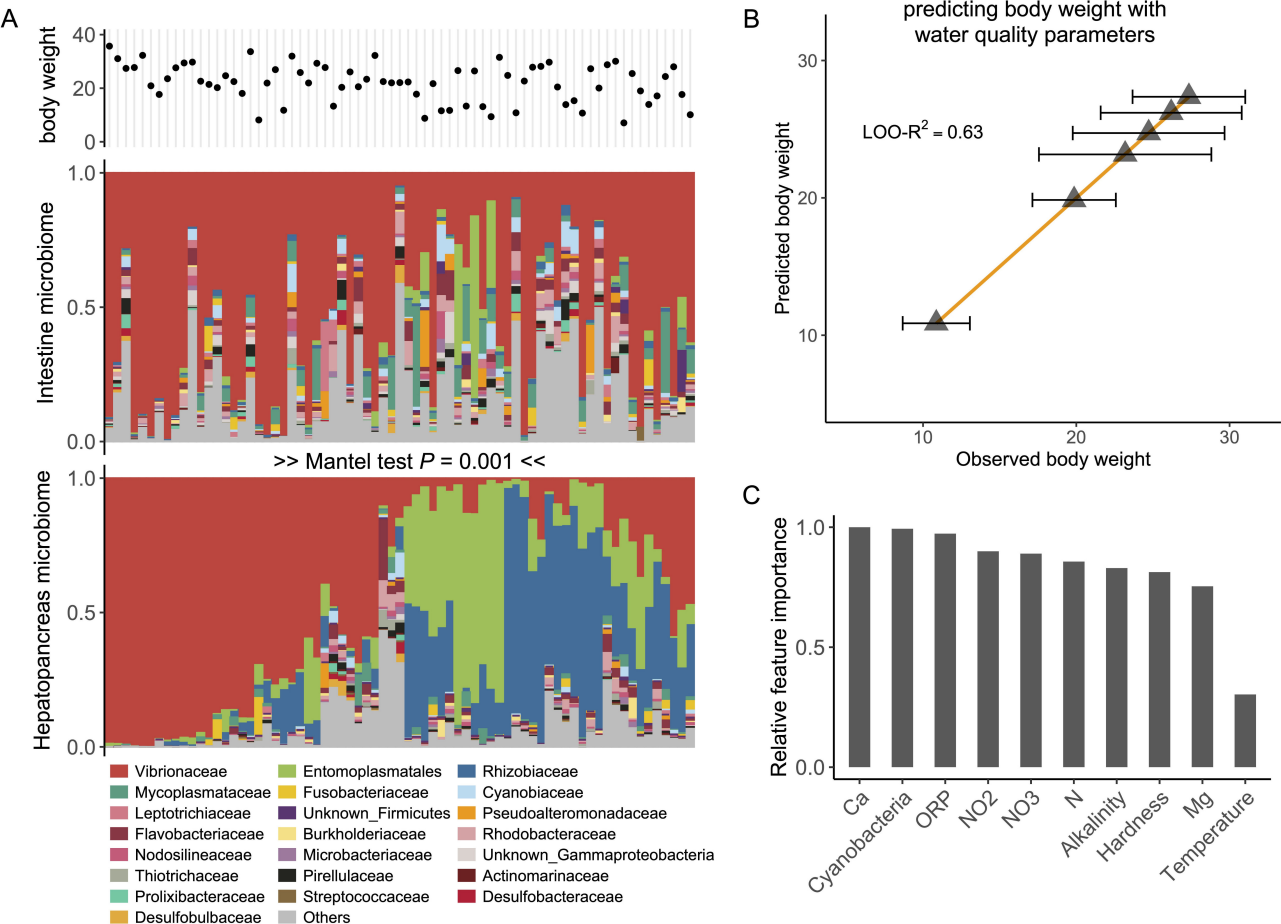
Adult shrimp body weight is better predicted by water quality than microbiome composition. (**A**) Adult shrimp individuals were sampled for body weight measurement and microbiome sequencing of hepatopancreas and the intestine. The microbiome composition in the intestine and that in the hepatopancreas are significantly correlated with each other. (**B**) The body weight of adult shrimp can be accurately predicted by water quality parameters. (**C**) Relative feature importance of water quality parameters in predicting adult shrimp body weight. ORP, oxidoreductive potential.

The hepatopancreas data revealed a relatively simple community structure dominated by three clades: Vibrionaceae, Entomoplasmatales, and Rhizobiaceae (Fig. 5A). Notably, the representative sequences for Entomoplasmatales and Rhizobiaceae differed significantly from any known organisms (92% and 87% maximum sequence identity over the 16S rRNA V3-V4 region), and likely correspond to endosymbiotic bacteria. For the Entomoplasmata, we were able to assemble a genome from metagenomic reads, which was classified as Candidatus *Hepatoplasma crinochetorum*, an endosymbiont in non-insect arthropods (48). Again, this genome showed less than 80% average nucleotide identity with any previously sequenced organism, highlighting the presence of unknown bacterial taxa within the shrimp hepatopancreas. In contrast to the hepatopancreas microbiome, the shrimp intestine microbiome exhibits significantly greater diversity (Fig. 5A, ASV richness Wilcox test *P* < 0.001). Despite the distinct compositions of the intestine and hepatopancreas microbiomes, a significant correlation exists between them (Mantel test *P* < 0.001), suggesting a systemic linkage in microbial compositions across different digestive tract sections.

Based on this characterization of the hepatopancreas and intestine microbiomes, we sought to determine to what extent microbiome composition across the adult shrimp digestive tract could predict body weight in grow-out ponds. Our data revealed that the intestine and the hepatopancreas microbiome compositions were weak, although significant predictors of shrimp body weight (LOO-R^2^ = 0.16 for 226 samples with hepatopancreas microbiome; LOO-R^2^ = 0.18 for 76 samples with intestine microbiome). The observed R^2^ were similar to the levels observed in other livestock gut ecosystems (average cross-validated R^2^ <0.2) (49) and significantly different from those obtained with randomized data (LOO-R^2^ = 0.03 ± .01 for 100 random tests, *P* < 0.001). Moreover, variation in body weight could not be attributed to differences in alpha-diversity of either intestine or hepatopancreas microbiome (Fig. S5). In contrast to the high degree of unexplained variance across individuals, our analysis of the metadata showed that the average body weight of individual ponds correlates strongly with water quality parameters (LOO-R^2^ = 0.63 for 226 samples, Fig. 5B), indicating that the average animal weight is primarily controlled by water chemistry. Among the key factors revealed by the data, alkalinity, hardness, oxidoreductive potential, nitrogen concentration, and cyanobacteria density emerge as key predictors (Fig. 5C). Alkalinity, indicating buffer capacity of the pond water, is closely correlated with productivity in shrimp farms (50). Hardness is determined by the concentration of dissolved calcium and magnesium, which are important for exoskeleton development and osmoregulation (50, 51). Cyanobacterial blooms can have adverse effects due to toxin production and eutrophication (52). These factors highlight the complex dynamics between water chemistry, eutrophication, and animal health that control productivity, and thus feed efficiency, in large-scale aquaculture facilities.

## DISCUSSION

In this study, we found that microbiome composition alone explains approximately 50% of shrimp larvae survival—a remarkably high effect that underscores its potential as an early indicator of host health. This finding is especially striking given the data-limited conditions under which we are operating (Fig. 3C) and the additional variance likely contributed by factors such as host genetics, water quality, and farming practices. The microbiome associated with larvae likely affects host production by aiding in the breakdown of polymeric organic materials or by synthesizing beneficial growth factors such as vitamins, as suggested by our comparative genomics analysis. While prior research has demonstrated that mapping the structure to the function of microbial communities can facilitate functional predictions (53–55), these studies were primarily based on simplified microcosms in laboratory settings. Our investigation extends these findings by establishing a robust structure-function relationship within a complex, real-world ecosystem. Notably, our study reveals that tree-based models, like Random Forest and Gradient Boosting, largely outperform linear models in predicting functional output in this intricate real-world context (Fig. S7). This suggests that the functional landscape of natural microbiomes might be more complex than that of synthetic microbial communities with low biodiversity.

With respect to adult shrimps, which develop in less-controlled, complex ecosystems like grow-out ponds, our analysis shows that water quality parameters are strong predictors of the average shrimp weight per pond. However, there is a large degree of variance in individual body weight, which the microbiome can only partially explain (<20%) likely due to variable chemical and biological conditions in these environments. This suggests that hatcheries may be a better target for designing microbiome-based interventions during early developmental stages, especially considering that, as shown in our data, currently used probiotics based on non-native bacteria display low engraftment rates.

While this study highlights the predictive power of the microbiome for shrimp health at the larval stage, future research is needed to uncover the underlying mechanisms by which microbial species influence shrimp health. A key step in this endeavor is to move beyond taxonomic descriptions and develop a deeper understanding of the functional roles of microbial communities. Depending on their functions, microbes may be detrimental to shrimp health by producing toxins or virulence factors or beneficial by facilitating organic matter degradation or inhibiting pathogen growth. However, characterizing these functional roles is significantly more challenging than generating taxonomic profiles through sequencing. Addressing this challenge will require the development of experimental systems with genetic tractability and precisely controlled environmental conditions. For instance, comparative studies using wild-type and mutant microbial strains could help identify the genetic determinants responsible for harmful effects or beneficial effects. Importantly, these effects must be studied under environmentally relevant conditions, as microbial physiology and ecological interactions can be highly dependent on their environmental context (43, 56). Understanding these effects will be instrumental in predicting and improving shrimp health.

Our study uncovers an understudied taxonomic and functional diversity within the shrimp-associated microbiome, opening avenues for microbiome engineering. For example, a gene cluster involved in cofactor biosynthesis, prevalent across five genera of Rhodobacterales, shows strong signs of adaptive potential. Similarly, the widespread horizontal transfer of the TBDR gene among our isolates in Ecuador, as well as those from Asian aquacultures, suggests a universal fitness advantage for various strains of Flavobacteriales in widely distributed aquaculture systems. This points to the potential of engineering polysaccharide degradation-assisting probiotics (e.g., Flavobacteriales) that are adaptable across global shrimp aquaculture settings. These findings provide promising targets for downstream genetic and biochemical studies, paving the way for innovative strategies to optimize shrimp production by microbiome engineering.

## MATERIALS AND METHODS

### Sampling in the hatchery

Microbiome sampling was performed within a commercial hatchery on the coast of Ecuador from January 27 to February 19, 2021. A total of 130 tanks with *P. vannamei* were sampled at the end of the production cycle when the survival rate of shrimp larvae was also recorded. There were 13 tanks presenting an outbreak of disease, and samples of larvae from these tanks were collected. Furthermore, for a subset of 4 tanks, we sampled the shrimp larvae every 2 days throughout the developmental stages from nauplius (N), zoea (Z), mysis (M), and postlarvae (PL). This results in 9 timepoints spanning 18 days from N5 to PL10 stages for each tank. The shrimp larvae were fed with microalgae by the zoea stage (N5 and Z2 data points in our samples), and then *Artemia* since the mysis stage (M1 to PL10 data points in our samples). The feed microalgae, *Artemia*, together with the ambient seawater and administered probiotics were also sampled for microbiome sequencing to track the potential source of microbiome in the shrimp larvae.

### Sampling in shrimp grow-out ponds

A total of 224 adult shrimp were collected from six different pools from a large-scale shrimp farm located on the coast of Ecuador and stored in cryovials with DMSO. The work was carried out wearing KN95 masks and nitrile gloves in a sterile area to prevent contamination. Adult shrimps were collected before feeding began, starting at 7 AM and finishing by 10 AM. Animals were kept in buckets with aeration and water from their respective pools until their turn for collection. Each animal was weighed and photographed, after which they were taken to a sterile area for dissection of the intestine and the hepatopancreas. Half of the hepatopancreas was used for DNA sequencing and the other half for microscopic observation to record the degree of lipid vacuole fullness and changes in color that could indicate sickness. The remaining half of the hepatopancreas and intestine were immediately frozen in separate cryovials with liquid nitrogen and kept at −20°C until shipment.

Water quality was analyzed by measuring pH, salinity, temperature, total ammonium nitrogen, nitrite, nitrate, total nitrogen, phosphate, phosphorus, N:P ratio, alkalinity, calcium, calcium hardness, magnesium, magnesium hardness, potassium, Ca:Mg:K ratio, hydrogen sulfide (H_2_S), Redox potential, and turbidity. Algae-type quantification and identification were also performed by counting in a Neubauer chamber.

### DNA extraction and amplicon sequencing

DNA extraction of the shrimp larvae samples, including food (microalgae, Artemia), probiotic, and rearing water was performed with the Gentra Puregene Tissue Kit (QIAGEN) following the protocol provided by the manufacturer, except that bead beating was used to increase the extraction efficiency for 60 seconds at 5,000 rpm. In brief, the extraction procedure includes cell lysis (1:1 cell lysis solution), RNA removal (4 µL of RNAse A), protein precipitation (250 µL Protein Precipitation Solution), DNA precipitation (100% isopropanol), and purification (70% ethanol). Purified DNA was shipped to Argonne National Laboratory (Lemont, IL) for amplicon sequencing on an Illumina MiSeq targeting the V4 region of 16S rRNA using the 515F and 806R primers. Sequence-specific peptide nucleic acid (PNA) clamps were used to block the amplification of host-derived mitochondrial 16S sequences at the V4 region without affecting the amplification of bacterial 16S rRNA gene sequences (57). However, benchmark tests showed that eukaryotic 16S sequences accounted for only a very minor fraction (<5%) of the total amplified reads (Fig. S8), even in the absence of PNA clamps. Based on these results, we chose not to use PNA clamps for subsequent sequencing of hepatopancreas and intestine samples.

Shrimp hepatopancreas and intestine samples were torn into small pieces using tweezers. These fragments were further homogenized using a TissueLyser III (Qiagen) for 5 minutes at 25 Hz with PowerBeads Pro plates (Qiagen). Following this, DNA extraction was carried out on the resulting turbid liquid using the DNAdvance Kit (Beckman Coulter), according to the manufacturer’s protocol. Briefly, the extraction process included cell lysis using a 1:1 cell lysis solution, DNA binding to magnetic beads, and purification with 70% ethanol. Amplicon sequencing was performed at Seqcenter (Pittsburg, PA) with Zymo Quick-16S Plus Library Prep methods to amplify the V3/V4 region with 341F and 806R primers. Illumina sequencing was performed on an Illumina NovaSeq X Plus sequencer in one or more multiplexed shared-flow-cell runs, producing 2 × 151 bp paired-end reads.

Amplicon sequence analyses were performed on the MIT Engaging computing cluster, where we used QIIME2 v2019.2 (58) to demultiplex the raw reads and the DADA2 plugin (59) to generate amplicon sequence variants (ASVs). Quality filtering and chimera removal were performed with the default parameters in DADA2. Briefly, sequences were trimmed to remove adapters, resulting in high-quality sequences with a quality score >38. This process yielded 9,729 ASVs for the hatchery stage samples and 10,426 ASVs for the grow-out stage samples. Taxonomy was assigned to representative ASVs using the classify-sklearn method in QIIME2 with the SILVA database v132. ASVs originating from eukaryotic 16S sequences were excluded before downstream analyses. The absolute ASV table output from DADA2 was normalized to relative abundances by dividing the count of each ASV by the total read count within the corresponding sample.

### Strain isolation and genotyping

We first isolated ~200 isolates using Difco Marine Broth 2216 media (BD). Colonies were isolated by first growing on agar plates prepared with MBL medium (60) containing homogenized Artemia as the sole carbon source, and then streaking to single colony purity on agar plates prepared with 1/10 diluted Marine Broth 2216 medium (adjusted for salt) containing 0.2% N-acetyl-glucosamine as the sole carbon source. A total of ~200 Rhodobacterales strains were further isolated with a customized media recipe favoring the growth of Rhodobacterales species among other marine copiotrophic heterotrophs (35). The media is MBL minimal media with 40 mM sodium succinate added as the carbon source. Marine Broth 2216 was spiked into the media with a dilution factor of 1:80 to facilitate bacterial growth. Vp was selectively isolated by the CHROMagar *Vibrio* (CaV) selective media, where the mauve colonies were picked for genotyping. Genotyping of all isolates was performed by full-length 16S Sanger sequencing (GENEWIZ at Azenta Life Sciences). 16S sequences of isolates were aligned to ASV sequences from the shrimp larvae microbiome with blastn in the blast suite v2.12.0. A maximal-likelihood phylogenetic tree of representative 16S sequences was constructed with MEGA v11.

### Whole-genome sequencing and genomic data processing

Shotgun whole-genome sequencing for representative isolates of Flavobacteriales and Rhodobacterales was performed at the SeqCenter (Pittsburgh, PA), on an Illumina NextSeq 2000 platform (2 × 151 bp pair-ended). Raw reads were trimmed to remove adaptors and low-quality bases (-m pe -q 20) with Skewer v0.2.2 (61). The remaining paired reads were checked for quality with FastQC v0.11.9. Quality-filtered reads were assembled into contigs with MEGAHIT v1.2.9 (62).

Nanopore long-read sequencing was combined with shotgun whole-genome sequencing also at the SeqCenter (Pittsburgh, PA) to close the genome of Vp. The closed genome was assembled using Unicycler v0.4.9 (63) by combining Illumina short reads and Nanopore long reads, resulting in two chromosomes and three circular plasmids. The assembly graph in gfa format was visualized by Bandage v0.8.1 (64). Coding sequences are predicted by prodigal v2.6.3, followed by functional annotation by eggnog-mapper v2 (65) (--go_evidence non-electronic --target_orthologs all --seed_ortholog_evalue 0.001 --seed_ortholog_score 60).

### Metagenomic sequencing and processing

We did shotgun metagenomic sequencing for six shrimp hepatopancreas samples (two samples with the highest relative abundance of Vibrionaceae, two samples with the highest relative abundance of Entomoplasmatales, and two samples with the highest relative abundance of Rhizobiaceae). Shotgun sequencing, including library preparation, was performed at SeqCenter (Pittsburgh, PA) on an Illumina platform. We performed a quality check for pair-ended fastq files with FastQC v0.11.9, after which low-quality bases were trimmed by Skewer v0.2.2 (61) (-m pe -q 20). Quality-filtered reads were first filtered with Bowtie2 v2.5.3 (--un-conc) to get rid of all the sequences aligned with the *P. vannamei* genome (NCBI RefSeq accession GCF_003789085). This resulted in the removal of 60.75% ± 1.07% reads in the samples. All the unaligned sequences were then used to assemble contigs with MEGAHIT v1.2.9. The reads that were unaligned to the *P. vannamei* genome were mapped back to assembled contigs with Bowtie2 (66) 2.5.3 to generate a coverage table. Metagenomic assembled genomes (MAGs) were generated with CONCOCT (67) v1.1.0 (-c 10000). Generated bins were evaluated for completeness and contamination using CheckM (68) v1.1.2. Taxonomic classification of MAGs was performed by GTDB-tk v2.1.0 (69). We were able to generate a MAG of *Vibrio fischeri* (completeness 100%, contamination 0.5%) and a MAG of *Hepatoplasma crinochetorum* (completeness 87.5%, contamination 0%) through this effort.

### Identifying core clades from global shrimp-associated microbiomes

We compiled a comprehensive global data set of amplicon microbiome sequences, specifically targeting the 16S rRNA V4 region with an approximate overlap of ~250 base pairs (bp). This overlap facilitates the downstream analysis of core microbial clades. We retrieved amplicon sequencing data in FASTQ format from the National Center for Biotechnology Information (NCBI) database. Using DADA2 (59) within QIIME2 (58) (version 2019.2), we processed samples from various studies, yielding 48,514 shrimp-associated amplicon sequence variants (ASVs) and 7,999 coastal seawater ASVs.

We utilized MAFFT (70) to align all ASV sequences compiled from global studies (version 7.245), followed by sequence trimming to uniform lengths of overlapping ~250 bp using TrimAl (71). To ensure high-quality alignment of the vast number of sequences, we initially conducted stringent alignments (--maxiterate 1000) focusing on ASVs from the most abundant taxonomic groups, including Vibrionales, Rhodobacterales, Flavobacteriales, and Alteromonadales. These aligned sequences were trimmed to uniform lengths, creating a “backbone” alignment. Subsequent sequences were integrated into this backbone using MAFFT’s --add function. We then merged this composite alignment with pre-aligned reference sequences from the SILVA database (72) (release 132, Nr99) to construct phylogenetic trees for each major clade. After a final trim with Trimal, the sequences were standardized to approximately 250 bp. We constructed the phylogenetic trees using FastTreeMP (73), employing the -nt, -gtr, and -gamma options, and visualized the trees with iTOL (74). Moreover, we conducted homologous clustering of all shrimp-derived sequences using MMseqs2 (-s 7.5 –min-seq-id 1) (75). This analysis supports the power-law distribution illustrated in Fig. 1D.

To identify core shrimp-associated clades, we applied three stringent criteria. First, a clade must show significant enrichment (adjusted *P* < 0.05, with Bonferroni correction) of shrimp-derived sequences, assuming a binomial distribution of these sequences across the entire phylogenetic tree. Second, the clade must encompass shrimp-associated sequences that are globally ubiquitous and present in at least 80% of sampled global shrimp cultures. Third, we required complete homology within each clade, selecting only those with zero branch length, indicating 100% sequence identity. Ultimately, five clades met these criteria for significant enrichment, global prevalence, and stringent homology. We assigned representative taxonomy to each clade based on the dominant taxonomy of the corresponding SILVA (72) reference sequences.

For the overarching phylogenetic analysis, we constructed a tree with all 56,513 ASV sequences derived from both shrimp and coastal seawater. We labeled each tree tip according to its source. The observed mean phylogenetic distance (MPD) between shrimp-associated and coastal seawater sequences was calculated. To assess the significance of our findings, we randomized the MPD calculations 1,000 times by reshuffling the tip labels. The net relatedness index (NRI) is calculated by the standardized effect size of the MPD, which is [oMPD−mean(rMPD)]/sd(rMPD).

### Identifying characteristic genes of shrimp-associated niche

We aligned the 16S rRNA gene sequences of our 501 isolates to the Flavobacteriales and Rhodobacterales ASV sequences in the microbiome. This resulted in four Flavobacteriales isolates and five Rhodobacterales isolates with 100% identical sequences with an *abundant* ASV in the shrimp-larvae microbiome (at least 1% relative abundance in any sample). These nine isolates spanned nine distinct genera, prompting us to retrieve all available genomes for these genera from the RefSeq database. This search yielded 394 genomes for Flavobacteriales and 222 for Rhodobacterales.

Subsequently, we conducted protein sequence clustering using MMseqs2 (75) (-s 7.5 -min-seq-id 0.5 c 0.5). This analysis produced a matrix, organizing proteins by rows and genomes by columns. We then refined our search to identify proteins universally present in our shrimp larvae isolates yet scarcely found (<10%) within other genomes of the same genera. This rigorous filtering process highlighted eight genes meeting these specific criteria. We utilized EggNOG-mapper (65) v2 for functional annotation of the identified protein sequences. To visualize gene maps, we employed Clincker (76), facilitating a comprehensive representation of the gene distribution. Moreover, our extensive search within the public metagenomic database MGnify (77) led to the discovery of a 100% homologous sequence in mariculture environments in China.

### Machine learning-based predictions

We implemented a Random Forest (RF) model to predict outcomes like shrimp larvae survival rate and adult shrimp body weight based on microbiome composition, using leave-one-out (LOO) cross-validation for evaluation. In this method, the RF model, developed with the R package randomForest (78), is trained on all but one sample to predict the outcome for the excluded sample, a process repeated for each sample. Predictive accuracy is quantified by comparing observed and predicted values through R² from linear regression, with robustness checked against 100 randomized trials of the outcome vector. To assess how sample size affects prediction power, we varied sample subsets (*n* = 40–110, in increments of 10) and conducted LOO cross-validation 20 times for each size. We also compared RF performance against other machine learning methods like gradient boosting and lasso regression to gauge their efficacy (as detailed in Fig. S7), providing a comprehensive view of the predictive capabilities of various approaches within our data set.

### Other statistical analysis

All other statistical analyses are implemented with R (79) v4.1.3. The world map is visualized using the default world map in R package ggmap (80) v3.0.0.

## Data Availability

All raw data generated by this study have been deposited on NCBI under BioProject PRJNA1144440. This BioProject includes (i) amplicon sequencing data of shrimp larvae including its associated feed and environmental samples, (ii) amplicon sequencing data of hepatopancreas and intestine samples of adult shrimp, (iii) shotgun metagenomic sequencing data of selected adult shrimp hepatopancreas samples, and (iv) draft genomes assembled for the nine abundant isolates shown in Fig. 4. The ASV table and all ASV sequences we compiled from global aquaculture shrimp microbiome data set are publicly available at https://github.com/Xiaoyu2425/shrimp for unrestricted access. Detailed information and sequences of the horizontally transferred genes are provided in supplemental files S1 and S2.

## SUPPLEMENTAL MATERIAL

File S1 aem.02420-24-s0001.txt Genome region containing *pdxA, dapA*, and *rhmD* in ASV171 *Cribrihabitans* sp.

File S2 aem.02420-24-s0002.txt Genomic region containing TonB-dependent receptor in ASV72 *Psychroserpens* sp.

File S3 aem.02420-24-s0003.csv Number of reads mapping to ASVs for all samples.

File S4 aem.02420-24-s0004.txt 16S sequences of all isolates in this study.

Supplemental material aem.02420-24-s0005.docx Tables S1 and S2; Figures S1 to S9.

ASM does not own the copyrights to Supplemental Material that may be linked to, or accessed through, an article. The authors have granted ASM a non-exclusive, world-wide license to publish the Supplemental Material files. Please contact the corresponding author directly for reuse.
