# Supplementary material for: Microbiome determinants of productivity in aquaculture of whiteleg shrimp
Source: Appl Environ Microbiol. 2025 Apr 15;91(5):e02420-24. doi: 10.1128/aem.02420-24 (PMC12094023; doi:10.1128/aem.02420-24)
Supplement: Supplemental material — Tables S1 and S2; Figures S1 to S9. [file aem.02420-24-s0005.docx]

**Supplementary materials**

**Microbiome determinants of productivity in aquaculture of whiteleg shrimp**

Xiaoyu Shan^1^, Kunying Li^1^, Patrizia Stadler^1^, Martha Borbor^2^, Guillermo Reyes^2^, Ramiro Solórzano^2^, Esmeralda Chamorro^3^, Bonny Bayot^2,4^, Otto X. Cordero^1*^

1 Department of Civil and Environmental Engineering, MIT

2 Centro Nacional de Acuicultura e Investigaciones Marinas, CENAIM, Escuela Superior Politécnica del Litoral, ESPOL, Guayaquil, Ecuador

3 Universidade Federal de Santa Catarina, Brazil.

4 Facultad de Ingeniería Marítima y Ciencias del Mar, FIMCM, Escuela Superior Politécnica del Litoral, ESPOL, Guayaquil, Ecuador

* ottox@mit.edu

**File S1** Genomic region containing pdxA, dapA and rhmD in ASV171 Cribrihabitans sp. (Separate file attached).

**File S2** Genomic region containing TonB-dependent receptor in ASV72 Psychroserpens sp. (Separate file attached).

**File S3** Number of reads mapping to ASVs for all samples (separated file attached).

**File S4** 16S sequences of all isolates in this study.

**Table S1** List of microbiome studies contributing to our compilation of the global shrimp-associated microbiome.

| Site | #Sample | System | Type | Reference | accession |
| --- | --- | --- | --- | --- | --- |
| Dongying (Cdy) | 6 | shrimp | intestine | 18 | PRJNA411759 |
| Ningbo (Cnb) | 121 | shrimp | intestine | 19 | PRJDB3419 |
| Wenzhou (Cwz) | 6 | shrimp | intestine | 20 | Requested from the authors |
| Zhuhai (Czh) | 75 | shrimp | intestine | 21 | PRJNA542015 |
| Maoming (Cmm) | 41 | shrimp | intestine | 22 | PRJNA429671 |
| Wenchang (Cwc) | 101 | shrimp | larvae | 23 | PRJDB9538 |
| Vietnam (Vt) | 10 | shrimp | intestine | 24 | PRJNA422950 |
| Thailand (Th) | 48 | shrimp | intestine | 25 | PRJNA625419 |
| Malaysia (Ma) | 14 | shrimp | intestine | 24 | PRJNA422950 |
| Mexico (Me) | 23 | shrimp | intestine | 26 | PRJNA417739 |
| Ecuador (Ec) | 130 | shrimp | larvae | this study | N/A |
| Brazil (Br) | 4 | shrimp | intestine | 27 | PRJNA473127 |
| SW1 | 95 | seawater | seawater | 28 | PRJDB3419 |
| SW2 | 47 | seawater | seawater | 30 | PRJNA357334 |
| SW3 | 12 | seawater | seawater | 29 | PRJEB22038 |

**Table S2** The nine isolates from the clade of Flavobacteriales and the clade of Rhodobacterales whose corresponding ASV (100% sequence identity) are abundant in the original shrimp larvae-associated microbiome.

|  | GDTB taxonomy | maximal relative abundance |
| --- | --- | --- |
| ASV7_Flavobacteriales | *Meridianimaribacter flavus* | 0.24 |
| ASV14_Flavobacteriales | *Tenacibaculum singaporense* | 0.09 |
| ASV26_Flavobacteriales | *Muricauda* sp. | 0.03 |
| ASV72_Flavobacteriales | *Psychroserpens* sp. | 0.16 |
| ASV12_Rhodobacterales | *Leisingera* sp. | 0.24 |
| ASV15_Rhodobacterales | *Donghicola* sp. | 0.14 |
| ASV17_Rhodobacterales | *Phaeobacter italicus* | 0.05 |
| ASV28_Rhodobacterales | *Sedimentitalea* sp. | 0.07 |
| ASV171_Rhodobacterales | *Cribrihabitans* sp. | 0.01 |

**Figure S1** Enrichment of microbial taxa in coastal seawater microbiome and shrimp-associated microbiome. Black dots indicate significant enrichment in either shrimp-associated or coastal seawater microbiome (fold change > 2 and adjusted P value < 0.05).


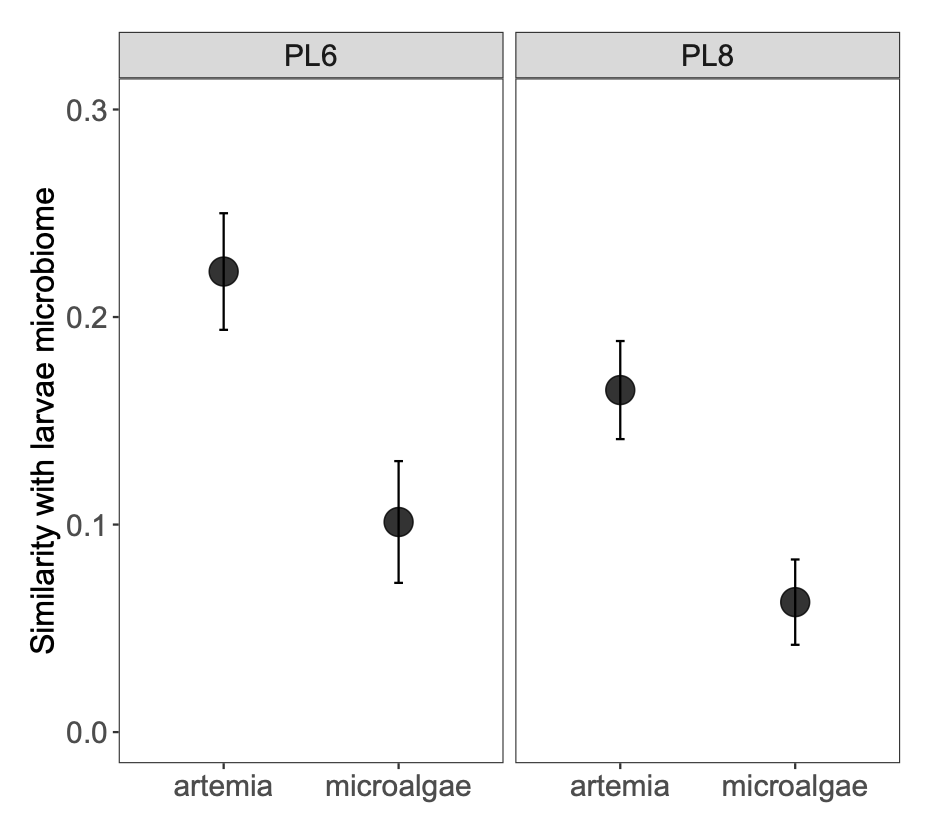


**Figure S2** At PL6 and PL8 stages, feed artemia has larger impact on the larvae microbiome composition than feed microalgae. All artemia and microalgae samples that are fed before PL6 or PL8 stages were used to calculate similarity with the microbiome composition of shrimp larvae microbiome at the respective stage.

**Figure S3** Relative abundance of Vp in larvae and feed microbiome in other three tanks where we temporally sampled across the developmental stages. Black line indicates richness of shrimp larvae-associated microbiome across developmental stages. Red bar and yellow bar indicate relative abundances of Vp across developmental stages in shrimp larvae microbiome and microbiome in the feed.


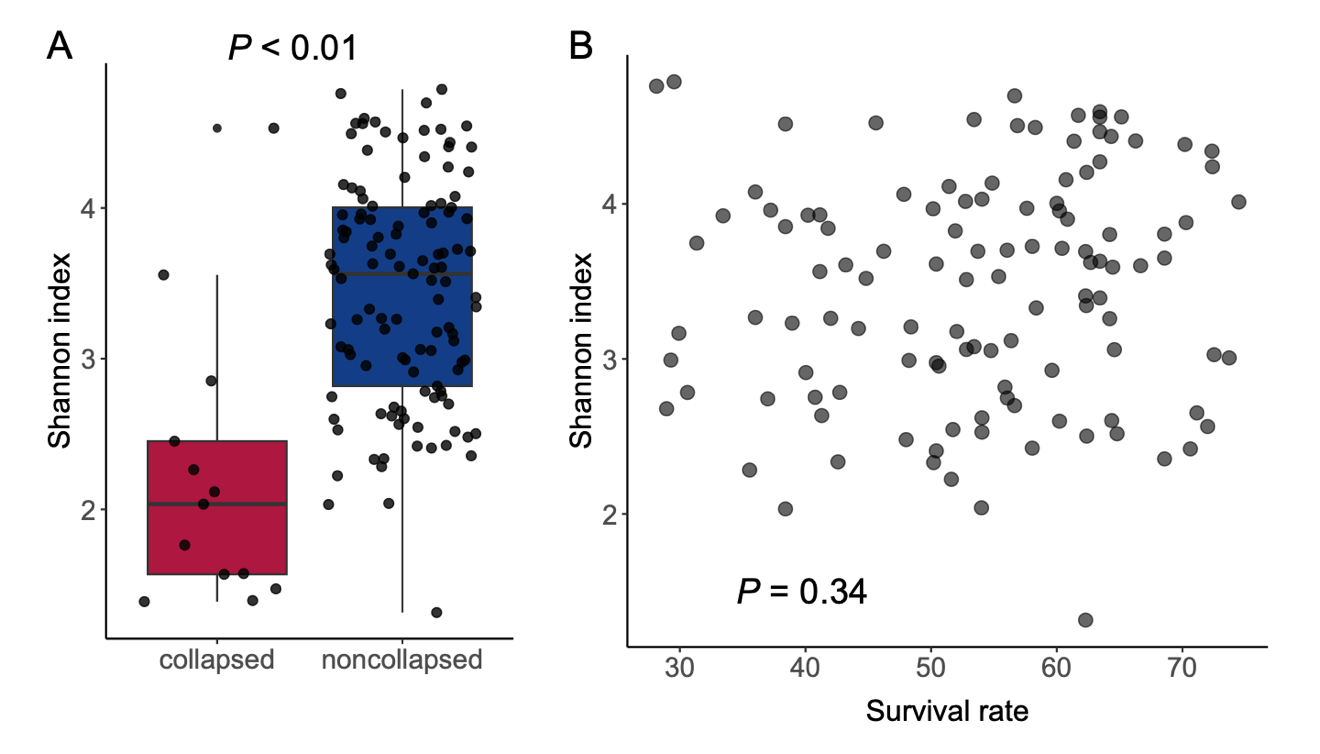


**Figure S4** Alpha diversity analyses of shrimp larvae microbiome. (A) Collapsed tanks driven by a surge of Vp show significantly lower Shannon index compared to those non-collapsed tanks (Wilcox test *P* < 0.01). (B) However, across those non-collapsed tanks Shannon index is not correlated with survival rate of the shrimp larvae (Pearson’s correlation test *P* = 0.34).


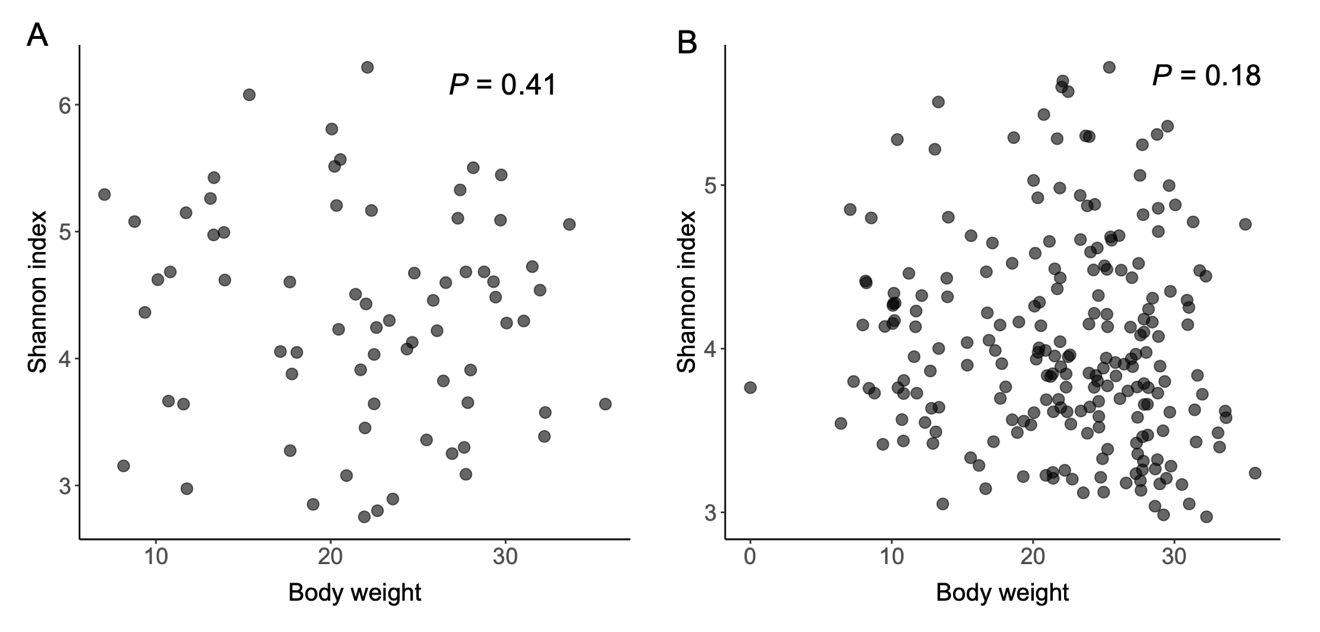


**Figure S5** Alpha diversity is not significantly correlated with body weight in grow-out ponds. (A) 76 samples of adult intestine microbiome and (B) 226 samples of adult hepatopancreas microbiome.

**Figure S6** Phylogenetic tree of 9 isolates in Flavobacteriales and Rhodobacterales with 100% identical sequences with an *abundant* ASV in the shrimp-larvae microbiome (at least 1% relative abundance in any tanks).

**Figure S7** Predicting shrimp larvae survival rate by microbiome composition, using different models (Random Forest, Gradient Boosting and LASSO regression).


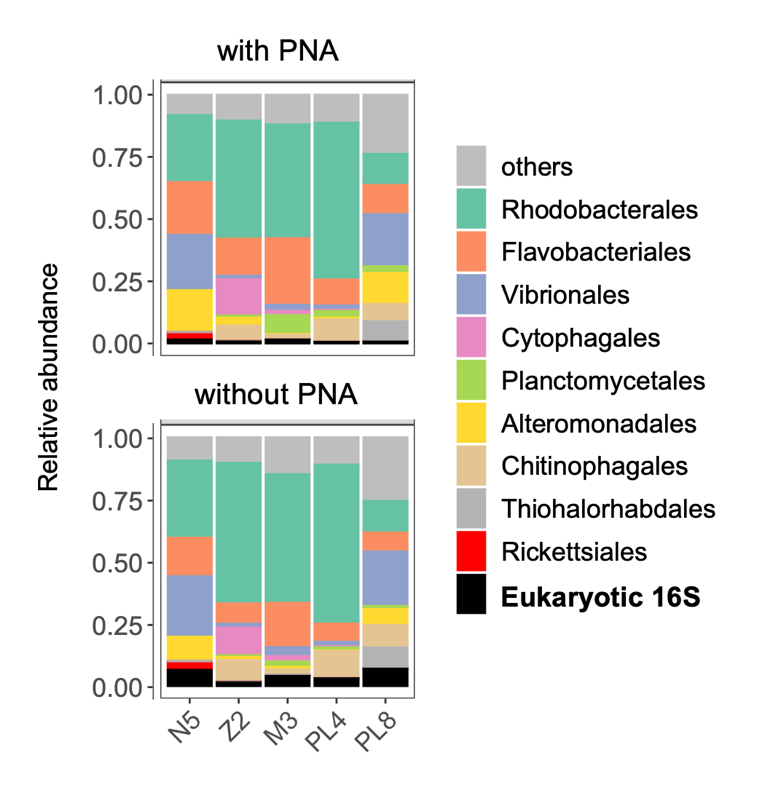


**Figure S8** Benchmark sequencing tests, conducted with and without PNA blockers, revealed that eukaryotic 16S sequences represent only a minor fraction of the total microbiome, even in the absence of PNA. This demonstrates the high specificity of the 16S primers used for amplifying bacterial 16S sequences.

**Figure S9** Alpha-diversity statistics of global shrimp microbiome samples. (A) ASV richness by recruiting different number of samples. Each dot represents the mean richness of repeating 100 times of random sampling. Error bar represents standard error, which is smaller than the visible scale in the plot. (B) Shannon index by recruiting different number of samples. ach dot represents the mean richness of repeating 100 times of random sampling. Error bar represents standard error. (C) ASV richness of each sample by the location of samples. (D) Shannon index of each sample by the location of samples.
